# Results of digitised blood smear differentiations by veterinary students using item analysis

**DOI:** 10.1038/s41598-024-84881-4

**Published:** 2025-02-18

**Authors:** Hannah Marahrens, Fritjof Freise, Frederik Kiene, Martin Ganter, Matthias Gerhard Wagener

**Affiliations:** 1 Clinic for Swine and Small Ruminants, Forensic Medicine and Ambulatory Service, University of Veterinary Medicine Hannover, Foundation, Hannover, Germany; 2 Department of Biometry, Epidemiology and Information Processing, University of Veterinary Medicine Hannover, Foundation, Hannover, Germany

**Keywords:** Medical research, Haematological diseases

## Abstract

Familiarisation with manual blood examination methods and the morphologies of leukocytes in peripheral blood contributes to routine veterinary practice. It enables veterinarians to verify automated analysis results and to examine blood cell morphology using the microscope. Third-year students therefore participated in an online module including 10 clinical cases of various mammal species with a haematological focus. Each case required the differentiation of 100 leukocytes using digitised cell images (= items) photographed from corresponding blood films. The study aims to provide insights into student difficulties with different leukocyte morphologies by calculating the Difficulty Index (DI) values. Out of 247 participating students, 96% completed the course in full, contributing 2197 differential white blood count (dWBC) responses for evaluation. The mean DI for all items (*n* = 1033) was 0.95 (± 0.09 SD), indicating overall low difficulty. Nucleated red blood cells (nRBC) (DI 0.98 ± 0.03 SD), segmented neutrophils (0.98 ± 0.07), and lymphocytes (0.97 ± 0.05) obtained high scores, whereas DIs for myelocytes (0.72 ± 0.14) and monocytes (0.82 ± 0.20) posed a greater challenge for the students to recognise these types of cells. Basophils, metamyelocytes, band neutrophils, platelets, and eosinophils ranged between DIs of 0.83 (± 0.12) to 0.94 (± 0.08). In contrast to hands-on microscopy, this digital format provided valuable training to gain routine in leukocyte differentiation and presentation, particularly of uncommon cell types. These should, however, be reliably distinguished by the examiner from the more common cell types, as they usually have a relatively high clinical significance even if they occur in small numbers. Nevertheless, the lack of dynamic manual adjustments during the microscopic examination emphasises the need for hands-on microscopy in combination with a digital format.

**Supplementary Information:**

The online version contains supplementary material available at 10.1038/s41598-024-84881-4.

## Introduction

Navigating the variety of animal species with their diversity of physiological characteristics presents a distinct challenge in veterinary medicine. This is reflected in various educational areas, including basic organism-related subjects such as physiology and anatomy as well as more specialised fields like pharmacology and pathology. As part of routine veterinary practice, it is also important to consider species-specific expertise when it comes to the interpretation of laboratory findings and blood analysis. The shapes, sizes and structural characteristics of red and white blood cells can vary considerably between species^1–4^, still posing challenges even for modern automated analysing systems^5–8^. Manual techniques are important for the understanding of automated processes and also enable veterinarians to critically question measurement outcomes when circumstances require it^9^. At the University of Veterinary Medicine Hanover, Foundation (Stiftung Tierärztliche Hochschule Hannover = TiHo), third-year students participate in a weekly clinical pathology course (CPC), learning basic diagnostic skills taught by the lab of the Clinic for Swine and Small Ruminants during winterterm. The students are in their first of three clinical years after completing the pre-clinical phase and are exposed to the preparation and interpretation of laboratory findings for the first time at this point of their studies. In addition to the CPC, there are weekly lectures in general internal medicine thematising theoretical backgrounds. In response to the contact restrictions due to the COVID-19 pandemic, the institution digitised certain haematological aspects of the CPC resulting in an online learning module (Moodle course = MC) containing 10 clinical haematological cases on the Moodle learning management system (TiHoMoodle). The contents of the MC-cases have already been described in a previous study, which presents the background and structure of the course, the individual cases with their specific haematological focus and the diseases covered in detail^10^. Teaching leukocyte morphologies formed the basis of the MC, as the differential white blood count (dWBC) offers insights into characteristics of infections and inflammatory responses and, when combined with the red blood count (RBC), haematological disorders^9,11^. In addition, basic species-specific differences in mammalian blood cells were addressed on explanatory slides when the individual case included special morphological features. The creation of dWBCs using images of leukocytes on screen formed the digital adaptation of microscopy training. To evaluate the students’ understanding of different leukocyte morphologies and assess the difficulty of specific cell types, an item analysis as part of the classical test theory was used for statistical analysis. This technique has been established in educational and psychological research to evaluate the use of individual questions or tasks (“items”) within a test. The method combining different parameters allows the assessment of the items’ difficulty and enables to distinguish between different levels of knowledge or skills among the participants themselves^12–14^. The key aspect is the difficulty Index (DI), which reflects the proportion of participants who answered an item correctly in relation to the number of all answers. Items garnering low numbers of correct responses indicate a high level of complexity, while those with many correct responses indicate elevated simplicity with 1.0 as reachable value maximum^12–14^. After our previous study^10^, which provides an overview of the MC contents with overall results for acceptance and usage, the intention of this work is to present the students’ results related to the specific leukocyte morphologies presented in the MC. The challenges could deserve particular attention during teaching contents of haematology as varying shapes, sizes and structural features of white blood cells could increase the complexity of their identification and classification.

## Methods

### Course description

The following section focuses on the tasks associated with the white blood count (WBC) in the MC. The course^10^ consisted of 10 clinical cases from various mammal species, which are commonly encountered in German veterinary practice (Table 1). Thematic focus revolved exemplarily around practical and relevant haematological cases. As the participants dealt with blood cell morphologies and blood count interpretation for the first time during their studies, they were initially introduced to the different relevant leukocyte cell types and their associated basic morphological characteristics using exemplary images, which also served as wall placates during the CPC microscopy training classes (Supplementary material). For more in-depth information, the students were provided with further literature in the form of an access to a German standard textbook on basic laboratory diagnostics in online version^15^. The teaching materials provided by the MC were tailored to the information in the lectures, in which the basics of haematology were also taught in theoretical form. The individual cases began with the differentiation of 100 single cell-images of leukocytes using single-choice questions. Therefore, cells had been previously photographed with a microscope camera at 1000× magnification using specific technical settings and haematological staining as previously described^10^. The MC-creating veterinarian, as one of the course instructors with a specialised background in haematology, manually photographed a pool of more than 100 cells for each case and selected the cells according to the white blood cell differentials of each virtual patient. This was done in consultation with the haematology laboratory staff which routinely work in the clinic’s laboratory in cases of uncertainties. Not all items of the total of 1033 cells could have been reviewed due to time limitation during the onset of the COVID-19 pandemic, therefore a few cells were only classified by one person. Cells were not selected according to specific criteria other than meeting basic morphologic recognition criteria for different types of leukocytes of mammal cells as thought in the accompanying lectures and the number of cells in the corresponding blood count. As the participants were dealing with blood cell morphologies for the first time, the aim was to select recognisable cells. All cases and the animal species with corresponding findings of the white blood cell differentials and the diseases addressed are listed in Table 1. The arrangement of presented cells during the differentiation task was randomised for each student and each attempt, with consistent answer options including all cell types relevant to the specific disease and case. In subsequent cases, the cell types were extended to include other cells found in blood smears, also allowing differentiation of nucleated red blood cells (nRBC) as immature, nucleated erythrocytes and the recognition of platelets and aggregates of these. Both are common sources of inaccuracies in automated counters^16–18^. After differentiation, students also calculated absolute leukocyte numbers in G/L (Giga per litre = 10^9^/L) and consequently the outcomes of the dWBC. The calculation task also remained uniform across the cases. In some cases, increased total leukocyte counts due to nRBC needed mathematical correction^10^, as being thematised in another task of every case, in which it has been a factor.

**Table 1:** Contents of the clinical cases included in the Moodle-course (MC) with the animal species, the mean findings in the white blood count (WBC), the peculiarities in the corresponding blood smears and the diseases addressed by each case. Modified table as partly published by Marahrens et al.^10^ in 2023.

Case	Species	Findings in WBC	Peculiarities	Diseases addressed
1	Donkey	Lymphocytosis	No further tasks to facilitate the introduction of the case principle	Chronic infectious disease of the respiratory tract
2	Sheep	Leukocytosis, neutrophilia	Hypersegmented neutrophils (more than five segments) = nuclear right shift^24^	Chronic purulent inflammation
3	Swine	Leukocytosis, lymphocytopenia, neutrophilia	Band neutrophils, metamyelocytes, myelocytes, transitional forms, = nuclear left shift^24,25^ Normoblasts	MMA (metritis, mastitis, agalactia) Iron deficiency, anemia Birth-related anemia
4	Goat	Leukocytosis, lymphocytosis, neutrophilia, basophilia	Nuclear right shift Small size of erythrocytes Form deviation of most erythrocytes Increased MCHC Decreased MCV	Basophilia in connection with allergic or parasitic reactions Spindle (like Sickle) cell anemia in goats
5	Alpaca	Neutropenia	Finely granulated neutrophils, metamyelocytes, myelocytes, transitional forms = nuclear left shift Normoblasts Oval shapes of erythrocytes	Acute enteritis Neoplastic changes
6	Swine	No deviation in the leucocytes from reference values	Changes in erythrocytes, Howell-Jolly-bodies, basophilic stripping Basophilia, normoblasts, aggregation of PLTs	Iron deficiency anemia Bleeding anemia *Mycoplasma suis* infection
7	Sheep	Leukocytosis, neutrophilia, eosinophilia Regenerative, normochromic, normocytic anemia	Eosinophilia	Parasitic infection of grazing ruminants, such as haemonchosis or fasciolosis
8	Reindeer	Neutropenia Regenerative, normochromic, normocytic anemia	Inclusion bodies (morulae) in granulocytes Howell-Jolly-bodies Azurgranulation in lymphocytes	*Anaplasma phagocytophilum*
9	Dog	Leukocytosis, neutrophilia, monocytosis	Nuclear left shift.	Monocytosis in dogs Pyometra
10	Cat	Leukocytosis, eosinophilia, monocytosis	Howell-Jolly-bodies in cats Morphologies of erythrocytes and thrombocytes in comparison to dogs	Eosinophilia in cats Gastrointestinal helminths in cats

### Cell images and answer options

A total of 1033 cell images were uploaded as individual items to be morphologically assigned in identifications. Table 2 presents the distribution of cell types across all cases. Segmented neutrophil granulocytes (Segmented neutrophils^19^) were the most frequently represented cell type with a total of 460 images and were predominantly presented in case 2, with 88 out of 100 items. Lymphocytes followed with 301 images, with case 1 having the highest number with 63 items. Band neutrophils^19^ were less frequent, representing a total of 93 images, with case 5 including the highest number with 40 items. Platelet aggregates and myelocytes were encountered infrequently. Both cell types were represented by 8 items each. The diversity of cell types between the cases ranged from 4 to 8 different cell types to be distinguished. Cases 1 and 2, as forming the beginning cases, included 4 different cell types each. Case 3 demonstrated the highest variety, encompassing 8 distinct cell types.

**Table 2:** Overview of the items of the course. Number of leukocyte images (items) occurring in the cases sorted according to their frequency in total, but also specified for each case. Modified from previously published table 1 in course description^10^. *nRBC* nucleated red blood cells.

		Case									
	Sum	1	2	3	4	5	6	7	8	9	10
Number of completed trials	2197	221	226	209	226	214	216	228	219	214	224
Number of items	1033	100	100	102	100	101	122	100	101	105	102
Answered correctly by all	265	16	52	0	32	5	35	40	16	40	29
In %		16	52	0	32	5	29	40	16	38	28
Cell variety		4	4	8	5	7	7	5	6	7	6
Segmented neutrophils	460	33	88	20	39	13	46	56	30	73	62
Lymphocytes	301	63	10	11	50	24	47	18	59	4	15
Band neutrophils	93	0	1	35	0	40	4	0	0	10	3
Eosinophils	55	2	1	1	5	0	1	24	4	1	16
Monocytes	36	2	0	1	1	12	2	1	2	11	4
Metamyelocytes	35	0	0	26	0	9	0	0	0	0	0
nRBC	26	0	0	2	0	1	18	0	0	5	0
Basophils	11	0	0	0	5	0	0	1	5	0	0
Myelocytes	8	0	0	6	0	2	0	0	0	0	0
Platelet aggregates	8	0	0	0	0	0	4	0	1	1	2

In terms of answer options, cases 1 and 2 initially presented with six choices (lymphocytes, segmented neutrophils, band neutrophils, eosinophils, basophils and monocytes). For the remaining cases, the list of options was expanded to also include metamyelocytes, myelocytes and nRBC. Additionally, platelet aggregates were included in cases 6, 8, 9 and 10.

### Evaluation and statistical analysis

All students whose data were included in the analysis had agreed to the privacy statement. Statistical evaluation was conducted using Microsoft Excel software (Microsoft Excel for Office 365) and R (R Foundation for Statistical Computing, Vienna, Austria). During export of the datasets to Excel, all items were coded with “F” combined with the position of their upload (e.g., “F1” as first uploaded item in every case, “F2” as second uploaded item. “F” is replaced by “P” for better understanding in Fig. 2).

### Item analysis

The item analysis and corresponding figures were generated using R (R Core Team, 2023) and the package ggplot2^20,21^. The difficulty analysis initially focused on overall course results and was subsequently applied to the individual cell types. As the DI represents the percentage of correct answers, the total number of correct answers was divided by the total number of answers to each specific item. Only data from students who had completed a differentiation within a maximum of 60 min and who had answered at least 50% of the items per case remained in the data set. Since the students could retake a differentiation, only the first attempt was analyzed in cases of repetitions.

### Ethical statement

This study^10^, which involved data from live animals (1 dog, 1 cat, 1 reindeer, 1 alpaca, 1 goat, 2 pigs, 2 sheep, 1 donkey), was conducted in accordance with the ethical guidelines of the University of Veterinary Medicine Hannover, Germany. The research was approved as it addresses the animal welfare standards set by the University, the German Animal Welfare Act, and Directive 2010/63/EU of the European Parliament and Council regarding the protection of animals used for scientific purposes. The blood samples were collected solely for diagnostic purposes by the Clinic for Swine and Small Ruminants, Forensic Medicine, and Ambulatory Service at the University of Veterinary Medicine Hannover, Foundation, Germany. As a result, the use of these data did not require approval from the IACUC or authorization under Directive 2010/63/EU. As the study was not conducted on animals in the animal model, the ARRIVE guidelines 2.0 are not applicable to this study.

The study^10^ involving human participants was reviewed and approved by the Data Protection Officer of the university. The participants have been informed about the processing and publishing of their data at the beginning of the course with access to the data protection notice containing the EU General Data Protection Regulation of 2018 and had to give consent before being able to start the course. All results were anonymized and processed in accordance with the relevant data protection regulation of the university and with EU Regulation 2016/679 DSGVO.

### Conference presentation

Parts of this study have already been presented at a conference and are therefore available as conference abstract: 31st Annual Meeting of the Section “Internal Medicine and Clinical Laboratory Diagnostics” (InnLab), DVG (Deutsche Veterinärmedizinische Gesellschaft e.V.), 02-03-2023, Göttingen, Germany.

## Results

### General course results

Out of the cohort, 237 (96%) students fully completed the MC, collectively completed 2197 dWBC responses (= 244821 individual image recognitions) across all cases presented in the course. A total of 265 (25.65%) of all items (*n* = 1033) were correctly recognised by all participants. While in case 1 there were 16 of the requested items (16%) achieving unanimously correct identification, the number increased to 52 items (52%) in case 2. This was followed by case 7 and 9, each with 40 items (40%) with only correct answers, followed by case 6 with 35 only correctly answered items (28.69%). In case 3, none of the items received unanimously correct identification by the students. Table 3 provides an overview of the cell types presented as items in the cases and general results.

**Table 3:** Summary of the DIs (Difficulty Indices) calculated for every cell type occurring as item in the course. Mean (± standard deviation SD), median, minimum (Min) and maximum (Max) values are given. The cell types are sorted according to their mean DI from high (not difficult) to low (more difficult). *nRBC* Nucleated red blood cell.

Cell type	*N*	DI in mean (± SD)	Median	Min	Max
All items	1033	0.95 (± 0.09)	0.99	0.12	1.00
Segmented neutrophils	460	0.98 (± 0.07)	1.00	0.25	1.00
nRBC	26	0.98 (± 0.03)	0.99	0.89	1.00
Lymphocytes	301	0.97 (± 0.05)	0.99	0.58	1.00
Eosinophils	55	0.94 (± 0.08)	0.98	0.64	1.00
Platelets	8	0.92 (± 0.07)	0.92	0.84	1.00
Band neutrophils	93	0.90 (± 0.10)	0.93	0.46	1.00
Metamyelocytes	35	0.88 (± 0.05)	0.89	0.76	0.96
Basophils	11	0.83 (± 0.12)	0.85	0.62	0.96
Monocytes	36	0.82 (± 0.20)	0.87	0.12	1.00
Myelocytes	8	0.72 (± 0.14)	0.75	0.46	0.87

The low standard deviation (SD) of 0.09 around the mean DI of all items (0.95, median = 0.99) indicates a narrow spread of the difficulty scores between the cell types. Notably, 95% of the items had DIs above 0.78. An analysis of the DIs related to the 10 cases revealed the minimum of 0.87 (± 0.10) in case 3 and the maximum of 0.99 (± 0.04) in case 2. The median DI remained 1.00 for most of the cases except for cases 1, 3, 5 and 8.

### Cell type associated results

**Fig. 1:**
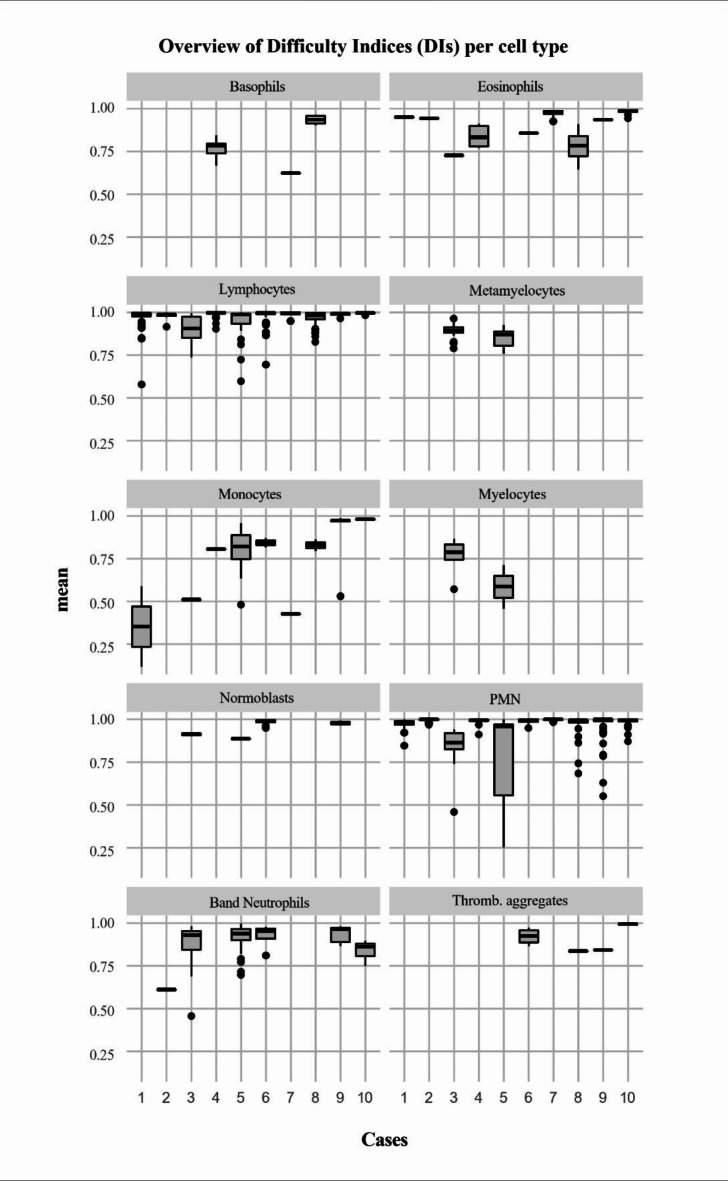
Box plots of the item difficulty indices (DIs) for the 10 cases, presented by cell type in alphabetical order as working result in R (R Core Team, 2023). The indices represent the proportion of correct responses to the items relative to the total number of items, with 1.00 as the possible reachable maximum. High values indicate items to be easy to differentiate, while low values indicate more difficulty. *DI* Difficulty Index, *PMN* Segmented Neutrophils, Thromb. Aggregates = Platelets.

The DIs for each cell type are presented in Table 3, sorted by the specific mean of cell types. Overall, the scores of the item characteristics of all items resulted in a mean DI of 0.95 (± 0.09 SD). The minimum of the item results was 0.12, the maximum 1.00. An overview of the scores depending on the cell type and on the cases is provided in Fig. 1.

While analyzing the dataset focusing on the specific leukocyte type, variations in DIs were observed:

#### Segmented neutrophils

The items showed a consistently high level of correct identifications across all cases with a mean DI of 0.98 (± 0.07) and a median of 1.00, while the minimum was 0.25 and the maximum 1.00. The lowest DIs, and therefore the highest difficulty, were reached in case 5 with a mean of 0.79 (± 0.25), followed by case 3 with a mean of 0.85 (± 0.11). In total, 41.1% (189) of all items showing segmented neutrophils were correctly identified by all participants. The highest difficulties were reached by items F2 in case 5 (DI = 0.25), F4 in case 3 (0.46) and F9 in case 5 (0.51).

#### Lymphocytes

Lymphocytes demonstrated a similar difficulty level with a consistently high level of DI with 0.97 (± 0.05) in mean and a median of 0.99, with 0.58 as minimum reached in case 1 and 1.00 in maximum of the items. The highest difficulty was reached in case 5 (mean DI 0.94 ± 0.10), while cases 4, 7 and 10 reached a mean of 0.99 (± 0.02, 0.02 and 0.01). Lowest values were achieved by F99 in case 1 (DI = 0.58), F65 in case 5 (DI = 0.60) and F44 in case 6 (DI = 0.70). 71 (= 23.6%) of all 301 items showing lymphocytes were correctly recognised by all students.

#### Band neutrophils

These presented a slightly higher difficulty with a mean DI of 0.92 (± 0.10) and a median of 0.93, with broader variations across the individual cases. The lowest DI in mean was reached in case 2 with 0.61, where one item (F89) presented this cell type. Case 10 reached the next lower DI with a mean of 0.84 (± 0.08), the highest DI being reached in case 9 with a mean DI of 0.93 (± 0.05). The lowest DI and therefore the highest difficulty was reached by F29 (DI = 0.46) in case 3, next to already mentioned F89 in case 1 and F54 (DI = 0.69) in case 3. None of the items reached the maximum of 1.00, i.e. none were correctly recognised by all students.

#### Eosinophils

Eosinophils also displayed an overall high level of identification, with a mean score of 0.94 (± 0.08) and a median of 0.98. The minimum DI was 0.64 and the maximum 0.996, as no item was answered correctly by all students. The lowest mean DI was reached in case 3 with a value of 0.73, where only one item represented that cell type. Case 10 reached the highest DI with a mean of 0.98 (± 0.01), followed by case 7 with a mean DI of 0.97 (± 0.02). The lowest individual DIs were reached by item F91 in case 8 with 0.64, F102 in case 3 with 0.73 and F90 with 0.75 in case 8. After case 3, the items in case 8 reached the highest difficulty with a mean DI of 0.78 (± 0.11).

#### Monocytes

Monocytes were among the more challenging leukocyte types to identify, with a total mean DI of 0.82 (± 0.20), a median of 0.87 and greater variations across cases. The lowest DI was reached in case 2 with a mean of 0.35 (± 0.33), where item F37 reached a DI of 0.12, while the second item in that case reached 0.59. Other more difficult items are represented in F94 in case 5 with 0.48 and F64 in case 3 with 0.51. The highest DI was reached in case 10 with 0.98 (± 0.00). As far as monocytes were concerned, there were several cases in which only one or two items represented this cell type in the blood smears: Cases 1, 3, 4, 6, 7 and 8.

#### Basophils

Basophils were present in 3 cases with a total of 11 items. With a mean DI of 0.83 (± 0.12) and a median of 0.85, they presented moderate difficulty in comparison, with variations across cases ranging from a mean DI in case 7 of 0.62 (*n* = 1) to 0.94 (± 0.03) in case 8 with *n* = 5. None of the items achieved only correct answers; maximum DI was reached in case 8 with 0.96, low DIs were reached by the items F100 with 0.62 in case 7, F98 with 0.67 in case 4 and F96 with 0.74 in case 4.

#### Metamyelocytes

Metamyelocytes, represented as items in case 3 and case 5, overall exhibited moderate difficulty with small variations between the cases. In case 3 a total of 26 items reached a mean DI of 0.89 (± 0.04), a median of 0.89 and a minimum of 0.79 with maximum 0.96. Case 5 reached a mean of 0.85 (± 0.06) with a total of 9 items, a median of 0.87 with a minimum of 0.76 and a maximum of 0.93. The individual lowest DIs were reached by item F62 with 0.76 and F55 with 0.78 in case 5.

#### Myelocytes

Myelocytes emerged as the most challenging leukocyte type to identify, with a mean DI of 0.72 (± 0.14) and a median of 0.75. In total this cell type was rarely represented, with 6 items in case 3 and 2 items in case 5. The lowest DIs were reached by item F63 in case 5 with 0.46, F60 with 0.57 in case 3 and F64 with 0.71 also in case 5. None of the items achieved totally correct answers, as the maximum was 0.87, reached by an item in case 3.

In cases of the non-leukocyte cell types, the difficulty reached lower levels, as the DI values were consistently high. NRBC showed a mean index of 0.98 (± 0.03) and a median of 0.99, the minimum was high with 0.89 (F101 in case 5) and the maximum was 1.00, while 4 items (= 15.4%) were correctly answered by all students. Platelets aggregates also reached high DIs and were therefore in lower difficulty levels to recognize, as the total mean DI was 0.92 (± 0.07), the median 0.92 and the minimum 0.84. One (= 12.5%) of all 8 items in total was correctly answered by all students (F101, case 10).

**Fig. 2:**
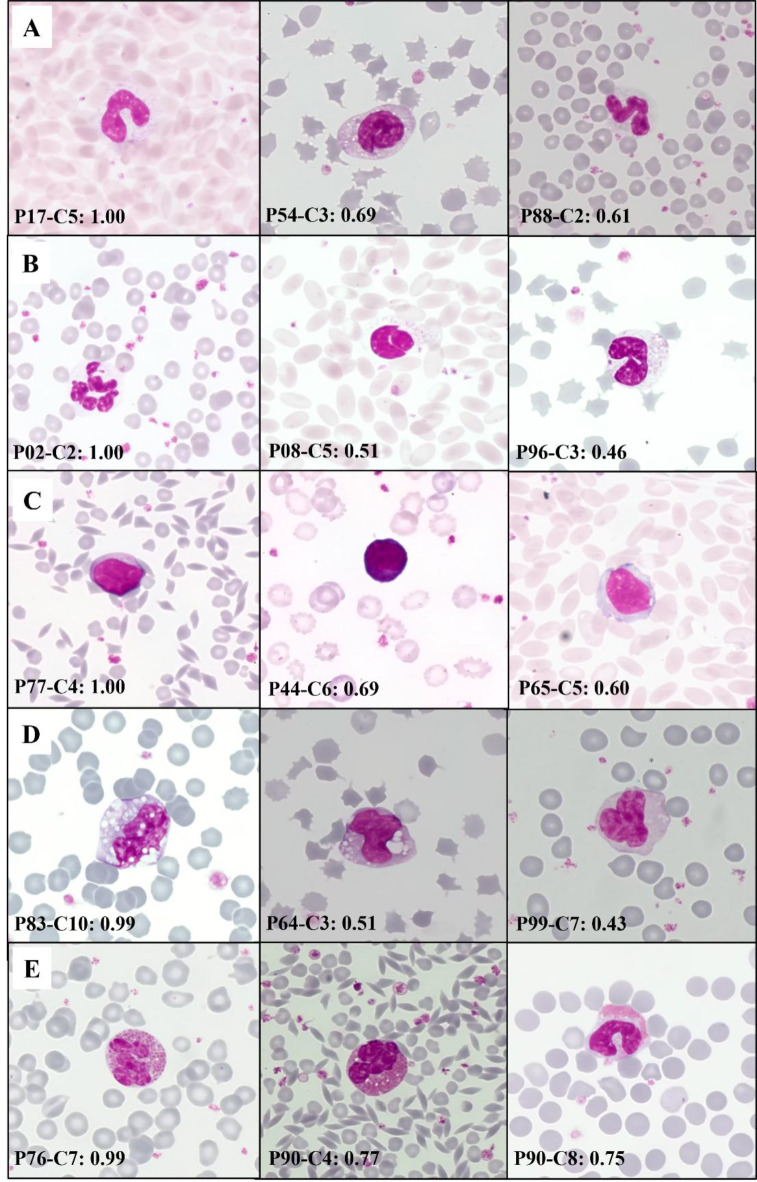
Collection of exemplary items of the course differentiated by the students representing typical leukocyte types occurring in blood films of mammals. Coding of each item represents the positioning (“P” = Photo) and the clinical case (“C” = Case) of the Moodle course, followed by the difficulty index value of the item. First items of each line achieved best values, while the other items of the same cell type appeared to be more difficult reaching lower DI values. In total, 1033 items were differentiated by each participant included in 10 clinical cases. Line A: Band neutrophils, B: Segmented Neutrophils, C: Lymphocytes, D: Monocytes, E: Eosinophils. Photos by M. G. Wagener.

A compilation of difficult items of all cell types can be seen in Fig. 2 with comparative examples of items that were correctly recognised by all students or achieved the highest DI values.

## Discussion

### Item analysis

In the context of this study, the difficulty indices (DIs) of the items provided a useful measure for assessing the students’ responses to the different leukocyte types in more detail. Generally, item analysis includes additional parameters such as the discrimination index or Cronbach’s alpha, which are used in combination with DIs to assess the quality of an item itself. The discrimination index would quantify items’ abilities to discriminate between students performing at higher or lower levels in a test, achieved through a comparison between the proportion of high-achieving individuals providing correct responses to the item to low-achieving individuals who exhibit similar accuracy^12–14^. Cronbach’s alpha quantifies the internal consistency or reliability as a value of how well each item in a group of items measures the same underlying concept and indicates how closely the items are related to each other within a cell type. Essentially, it assesses the extent to which all parts of the test contribute equally to the specific question. The values of Cronbach’s alpha range from 0 to 1, with higher values indicating greater reliability. It can also be used to verify the quality of the whole test instead of single items^12–14^. Items demonstrating elevated discrimination indices (> 0.4) are considered effective, as they differentiate between high and less achieving students^12^. When performing item analysis in our study, we only focussed on the difficulty index based on the consideration that the overall results are on such a high level that calculated values for the discrimination indices would not have been informative, as these are outcomes by comparing the number of correct answers from the top group (usually the top 27% or quartile) with the number of correct answers from the bottom group (usually the bottom 27% or quartile)^13^. A value close to 1 indicates high discrimination, which means that the item is useful to distinguish between high and low levels of students, while a value around 0 indicates that the item does not discriminate well^13^. The results for the discrimination indices would range around neutral in our case. As the aim of the study was not to assess the quality of the items, but the difficulty of the cell morphologies for students themselves, we consider the DI to be the appropriate technique.

### Leukocyte cell types

When examining leukocyte differentiation by veterinary students, the analysis revealed differences in difficulty between the specific cell types, while the overall difficulties resulted in generally low levels. Segmented neutrophils (DI 0.98 ± 0.07), lymphocytes (DI 0.97 ± 0.05) and nRBC (DI 0.98 ± 0.03) achieved the highest scores, indicating that these cell types were the easier cell types for students to differentiate (Table 3). As myelocytes achieved the lowest mean of 0.72 (± 0.14) in DI, this cell type seems to be the most challenging for students to identify, followed by monocytes with a mean of 0.82 (± 0.20) and basophils with a mean of 0.83 (± 0.12). Interestingly, Fig. 1 shows that the difficulties within the specific cell types were not distributed evenly across all cell types. For example, while comparing the graphics of the “easiest” cell types (segmented neutrophils and lymphocytes), the results for the individual items of the segmented neutrophils are more scattered than those of the lymphocytes, which were recognised more consistently at a higher level overall, but whose overall mean result is nevertheless lower than the result of the segmented neutrophils and lymphocytes therefore appear to be “more difficult” to recognise. Figure 1 also does not show linear improvement for the difficulty values within the individual cell types. As the individual cases varied in complexity from case to case, the study is not suitable to examine this aspect of development. The only exception is formed by the monocytes, where the index increased steadily with increasing case number. Monocytes also occur in various forms in peripheral blood smears but can still be recognised by the characteristic features such as vacuolated and basophilic abundant cytoplasm, variably formed nucleus and significant higher cytoplasm volume in relation to other blood cells^22^, so that their difficulty may have decreased with increasing practice in this course. It remains unclear why no similar development in difficulties can be observed in the other cell types. In case 5, the results for overall difficulty are particularly noteworthy. The variability of 7 different presented cell types (Table 2) is particularly high in this case, and an additional difficulty is the presence of leukocyte morphologies in process of maturation or activation, such as transitional stages between myelocytes, metamyelocytes, band and segmented neutrophils with partly nuanced different morphological features^23^. These transitional stages can be particularly difficult to distinguish and may have been mistaken. Both aspects are reflected in the results and are transferred in a more moderate form to the results of case 3. It is also striking that among specialised leukocyte types with conspicuous and typical characteristics, such as eosinophils^24^ or basophils^25^ with specific granules in cytoplasm, no item reached a maximum of DI 1.00, i.e. was correctly identified by all students by the end of the course. It could have been a problem that the focus could not be adjusted to take a detailed look at the cytoplasm. In the example of basophilic granulocytes, this could also be explained by their rare occurrence under physiological conditions and in the cases. Also, some of the demonstrated basophilic cells had only low numbers of granules in their cytoplasm, as one possible reason why they were individually difficult to differentiate, whereas eosinophils occurred in 8 of the 10 cases during the course. Considering these results, it is important to notice that different numbers of items represented the different cell types across the course, as there were 460 segmented neutrophils compared to 8 myelocytes for example (Table 2), so that at the end of the MC the students were trained differently in the various cell types. Reasons for different difficulties in the morphologies could be widespread. Figure 2 presents examples of items easy to identify with highest values compared to items of the same cell types which achieved lower DI. Line A shows band neutrophils, where P54-C3 shows a rolled-up nucleus, P88-C2 could be differentiated as segmented neutrophils, while P09-C5 in line B with segmented neutrophils and P96-C3 could have been differentiated as band neutrophils in the other way as for unexperienced participants it is difficult to imagine the cell nucleus dimensions in a cell during first attempts. The differentiation between band and segmented neutrophils can be challenging and was previously measured at the Institute by the width of nuclear bridges between the nuclear fragments: At the time of the course, it was taught that a neutrophil with a nuclear bridge narrower than one-third of the average nuclear diameter^26^ or the adjacent nuclear segments^27^ would be classified as segmented neutrophil, which is now considered obsolete. Other difficulties may have been the ratio of nucleus to cytoplasma volume, for example to differentiate between lymphocytes and monocytes. Diversity within a cell type could also be a common source of misdiagnosis when reactive lymphocytes develop a more basophilic and extensive cytoplasm, while P44-C6 as a small lymphocyte could have been mistaken for nRBC. Line D shows the diversity of monocytes with more or fewer vacuoles as sources of mistakes. One reason for a higher difficulty of P90-C8 in line E compared to P76-C7 could be the distribution and sizes of the eosinophil granules, which appear small and marginal in P90-C8. Still, the overall DIs range in high levels with a Minimum of DI = 0.72 (± 0.14) reached by the myelocytes as “most difficult” cell type. In comparison, Bichi and Embong (2018) described an acceptable range of difficulty index between 0.3 and 0.7, as exceeding values indicate the item of a test as being too easy^14^. Moeltner et al. (2006) presented the values 0.4 to 0.8 as a recommendation^13^. The overall high results for the DIs through all cell type suggest that students were able to correctly assign the cell images presented to them, indicating an understanding of the basic differences in leukocyte morphologies, also including different mammal species.

In the context of the clinical cases, students were confronted with different scenarios in blood smears, from the simple distinction between four cell types (cases 1 and 2) to more complex cases involving eight different possible leukocyte types (case 3). This variability represents another level of complexity, as in some cases certain leukocyte types are presented in excess, while, in other cases, the distribution is more variable, simulating clinical reality in which the prevalence of certain leukocytes can significantly vary. The students are expected to adapt their diagnostic approaches to align with these clinical variations, a skill that is more difficult to learn with reduced microscopy experience. Due to technical limitations of static image-based examination, such as the inability to change the focal planes while microscopy or other microscope settings to optimise visibility, students could face bigger challenges in assessing leukocyte morphology. This emphasises the need for direct interaction with microscopic specimens in veterinary education, a reason as to why training on a microscope should not be excluded from education. Furthermore, it is also plausible to suggest that digitising routine microscopy tasks could lead to more engaging and effective in-person teaching sessions, assisting students in developing both routine diagnostic skills and an understanding of diverse morphological features, which was one example of positive feedbacks provided by the students after finishing the MC^10^.

### Limitations

The course was designed from a didactic point of view as reaction on the sudden contact restrictions due to the pandemic onset and not to conduct this study, why the study settings are not ideal for elevation. The different cell types do not occur equally often, and the cases do not have a steady increase in difficulty. In addition, not all of the single cell photos were reviewed before being part of the course, as the creation of the course was under lack of time due to contact restrictions during the COVID-19-pandemic. This let to only a few items, which could not be classified reliably even by a group of experts and was considered to be acceptable in a total of 1033 items in general. Major limiting factors were that the assessment of leukocyte morphologies on the basis of cell images is impaired by the lack of ability for dynamic technical modification of the settings during the differentiation process. This includes the possibility of changing the acuity, the contrasts and the lighting conditions. The size of the visual field could not be adapted, whereas the image detail was relatively small. As such settings are manually adjusted on the microscope settings including the condenser, the microscope aperture and the objectives, training on how to manually adapt microscope settings should also be part of the training for veterinary students dealing with blood samples. Also, a voluntary feedback module for comments and suggestions by the participants at the end of the MC did not give information about individual challenges and difficulties while differentiating the individual cell types from each other. This leads to a lack of information about important factors future lecturers should consider when creating similar modules focussing on the distinguishing features of cell types and helpful mechanism to aid in their identification.

## Conclusion and outlook

The study found that veterinary students had varying degrees of difficulties in distinguishing leukocyte types, with some cell types proving more difficult than others. However, overall, the students showed a good understanding of the basic differences in leukocyte morphologies, which is consistent with the educational objective of teaching basic haematological identification skills. In addition, the study highlighted the potential of integrating digitising microscopy tasks into the student’s learning. In summary, the results highlight some complexity and challenges associated with leukocyte differentiation in veterinary haematology. To address these challenges, innovative teaching approaches that combine interactive digital methods with traditional hands-on experience could be relevant. As the majority of blood counts are generated automatically, it is necessary to manually check suspicious and obviously pathological blood counts by hand. In particular, the recognition of pathological cells is necessary and must be practised in context. While haematology education continues to improve, the development and evaluation of new teaching strategies is important to prepare future professionals for the wide spectrum of animal species in the field of veterinary medicine and changes in medical practice.

## Electronic supplementary material

Below is the link to the electronic supplementary material.

Supplementary Material 1

## Data Availability

The original dataset analysed and presented during the study are included in the article and are available from the corresponding author on reasonable request.

## Abbreviations

CPC: Clinical pathology course
DI: Difficulty index
dWBC: Differential white blood count
G/L: Giga per Litre (10^9^/L)
IRR: Inter-rater reliability
MC: Moodle course
nRBC: Nucleated red blood cells
RBC: Red blood count
SD: Standard deviation
TiHo: University of Veterinary Medicine Hannover, Germany
WBC: White blood count
