# Supplementary material for: Results of digitised blood smear differentiations by veterinary students using item analysis
Source: Sci Rep. 2025 Feb 18;15:5947. doi: 10.1038/s41598-024-84881-4 (PMC11836117; doi:10.1038/s41598-024-84881-4)
Supplement: Supplementary file 1 — Supplementary Material 1 [file 41598_2024_84881_MOESM1_ESM.pdf]

Lymphocytes

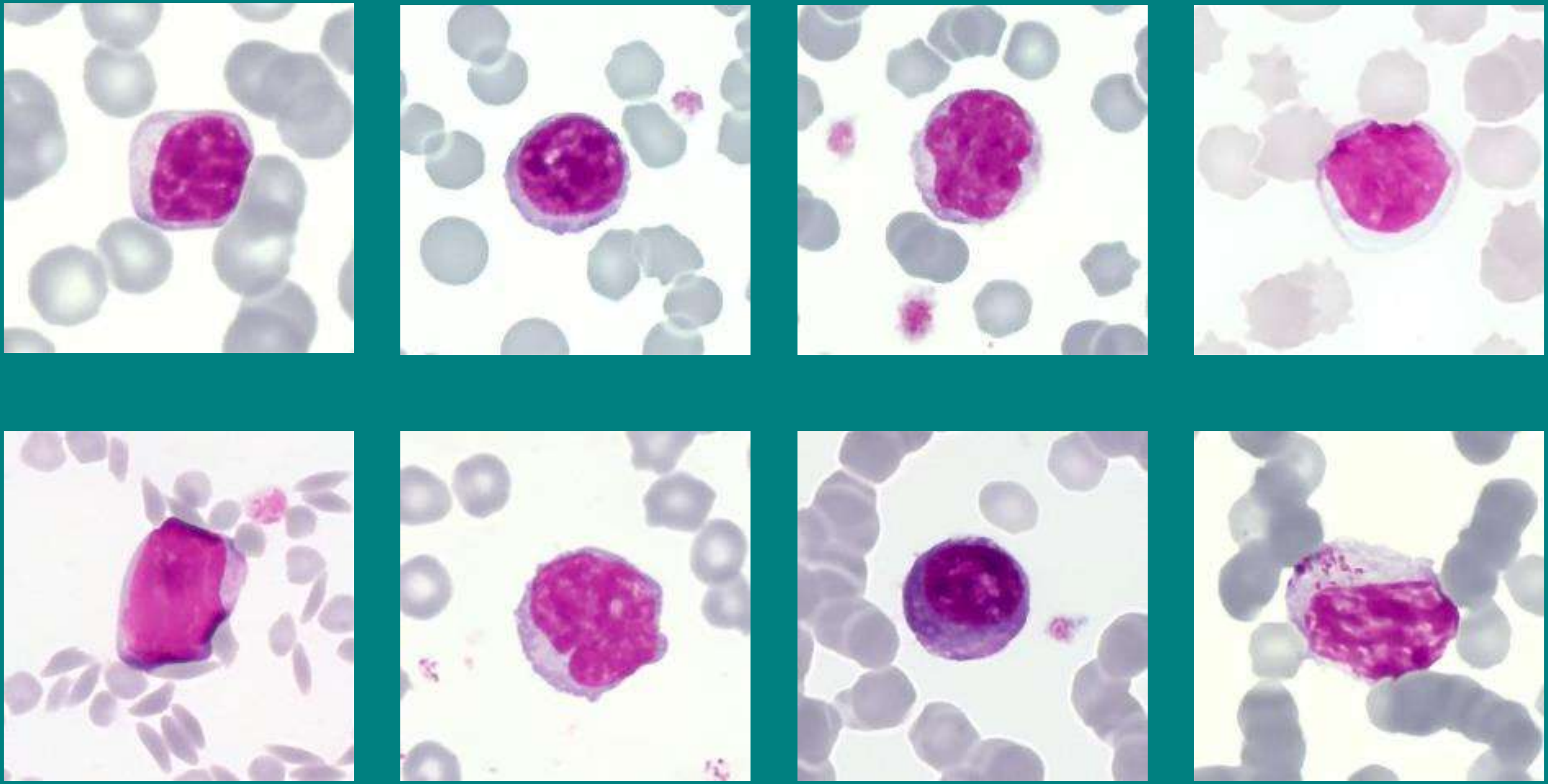

© M.G. Wagener, TiHo-Hannover, 2021

Monocytes

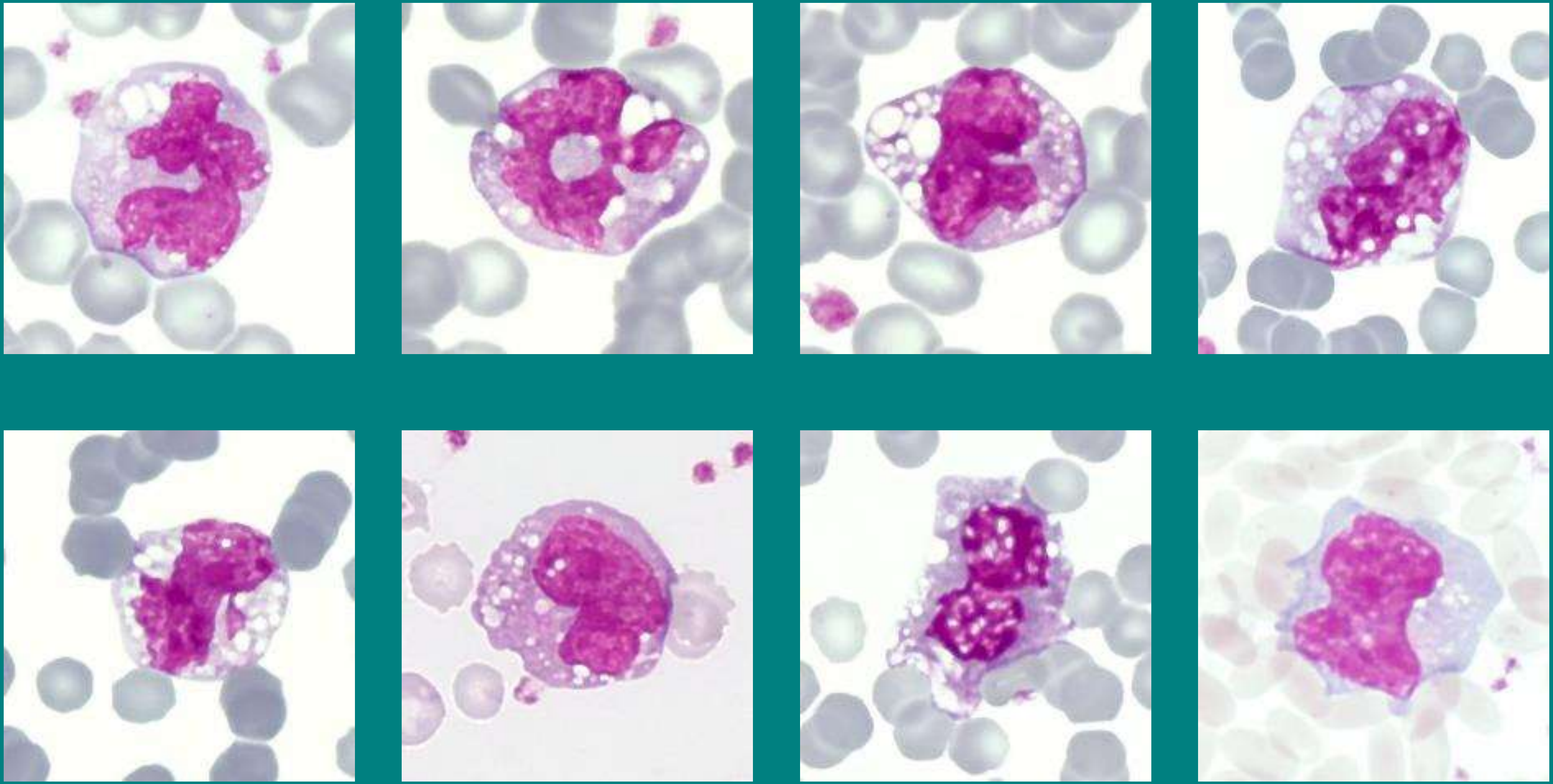

© M.G. Wagener, TiHo-Hannover, 2021

Segmented Neutrophils

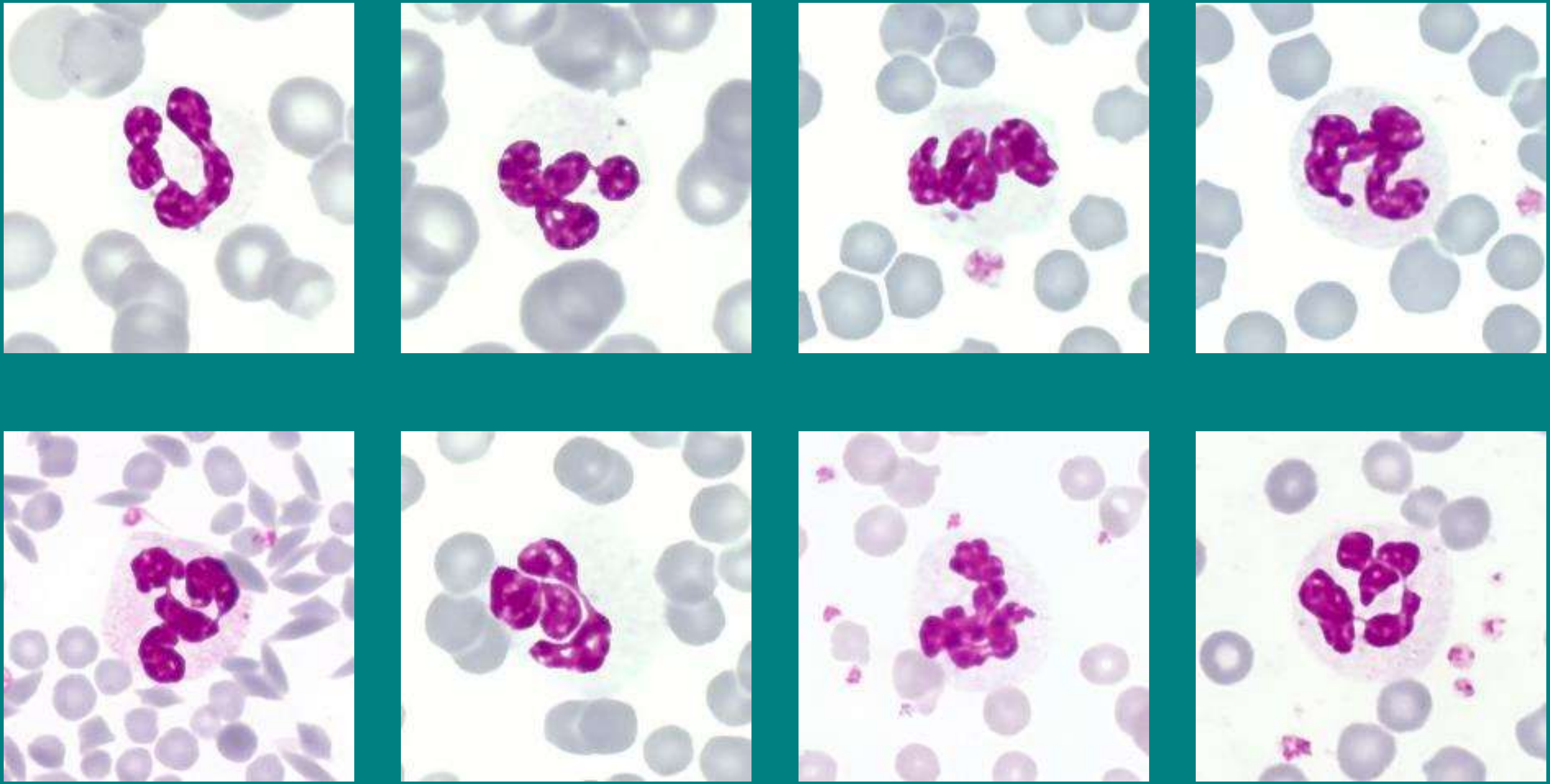

© M.G. Wagener, TiHo-Hannover, 2021

Band Neutrophils

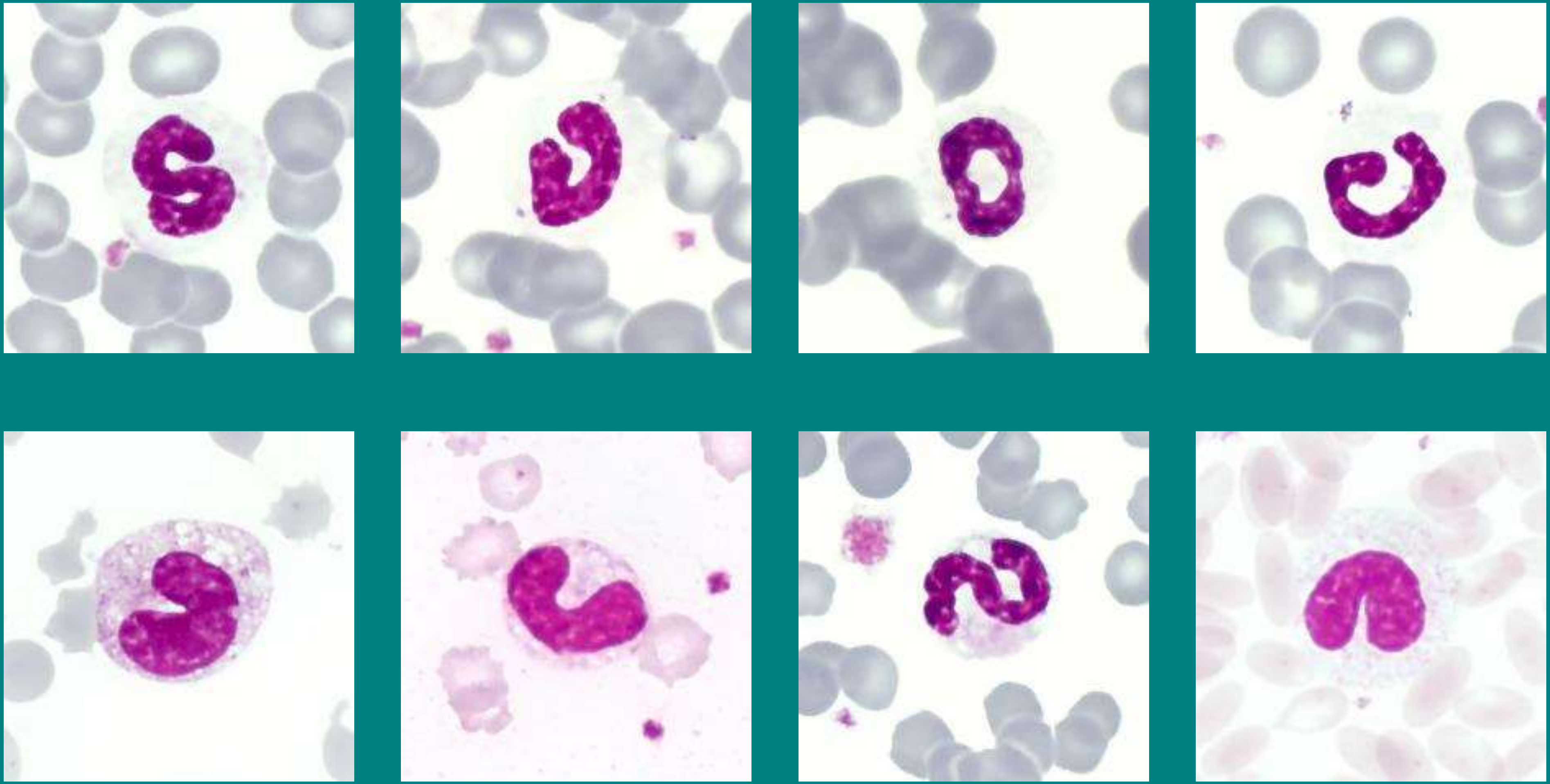

© M.G. Wagener, TiHo-Hannover, 2021

Eosinophils

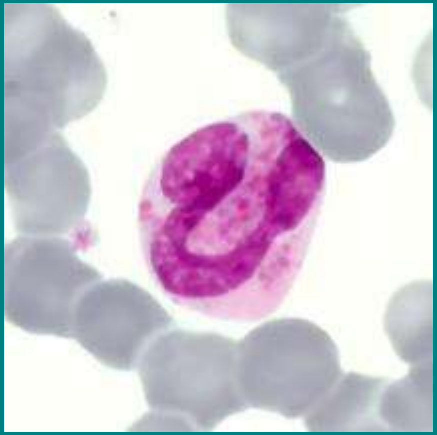

Dog

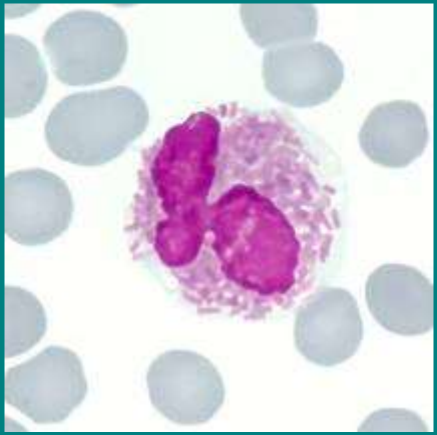

Cat

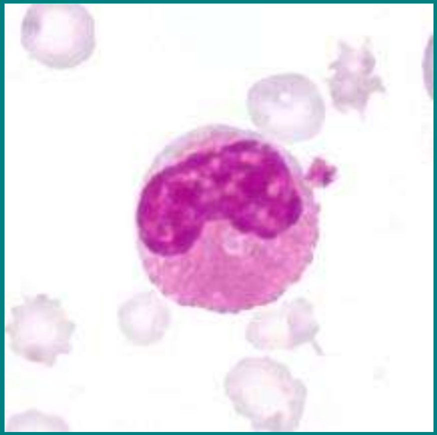

Swine

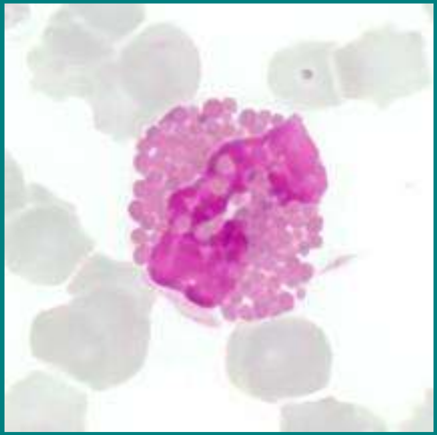

Donkey

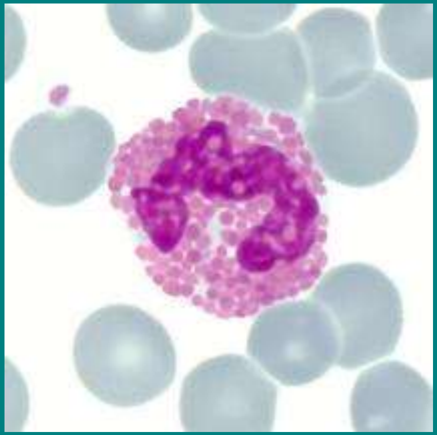

Rhino

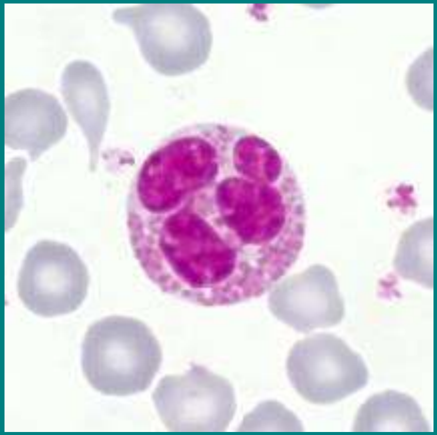

Sheep

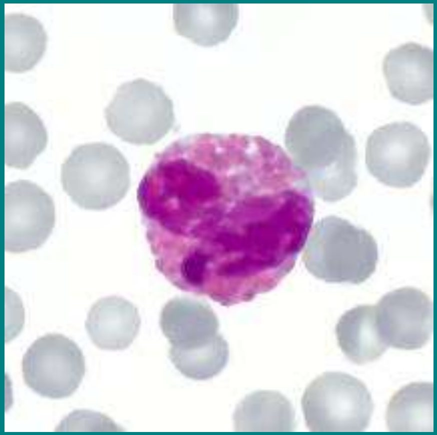

Antilope

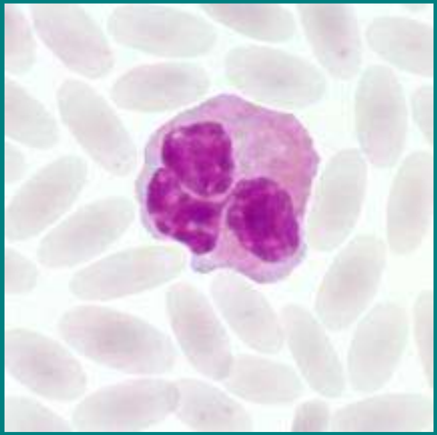

Vicugna

© M.G. Wagener, TiHo-Hannover, 2021

Basophils

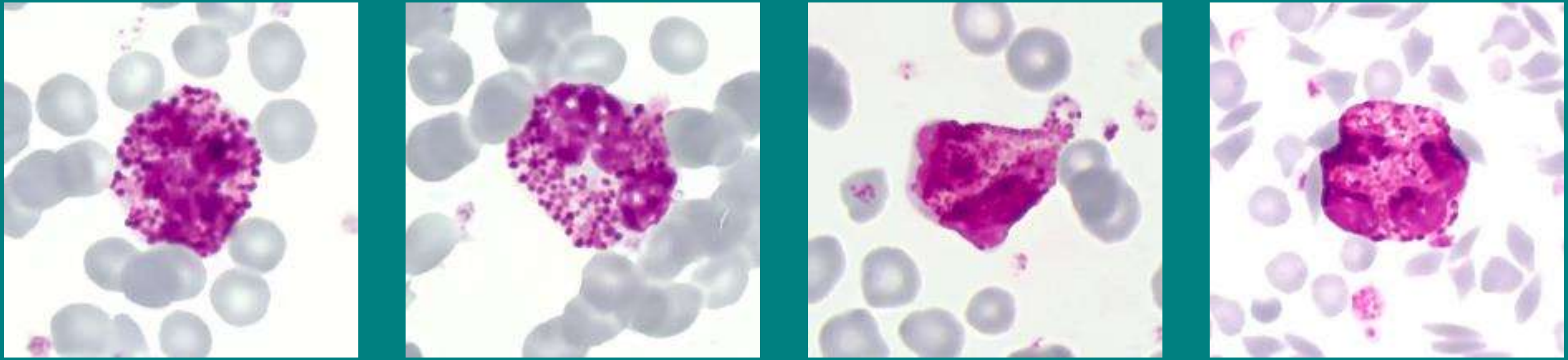

Platelets

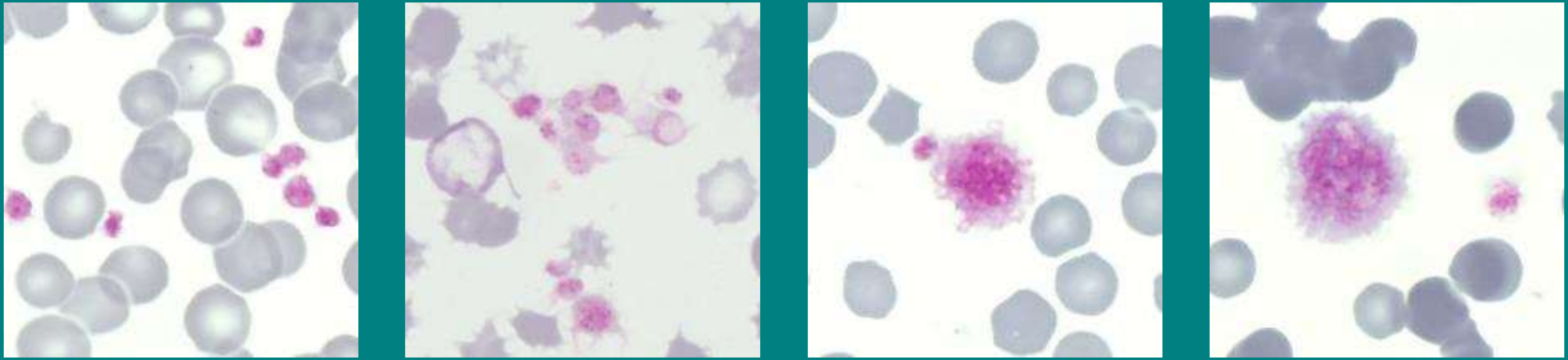

© M.G. Wagener, TiHo-Hannover, 2021

Metamyelocytes

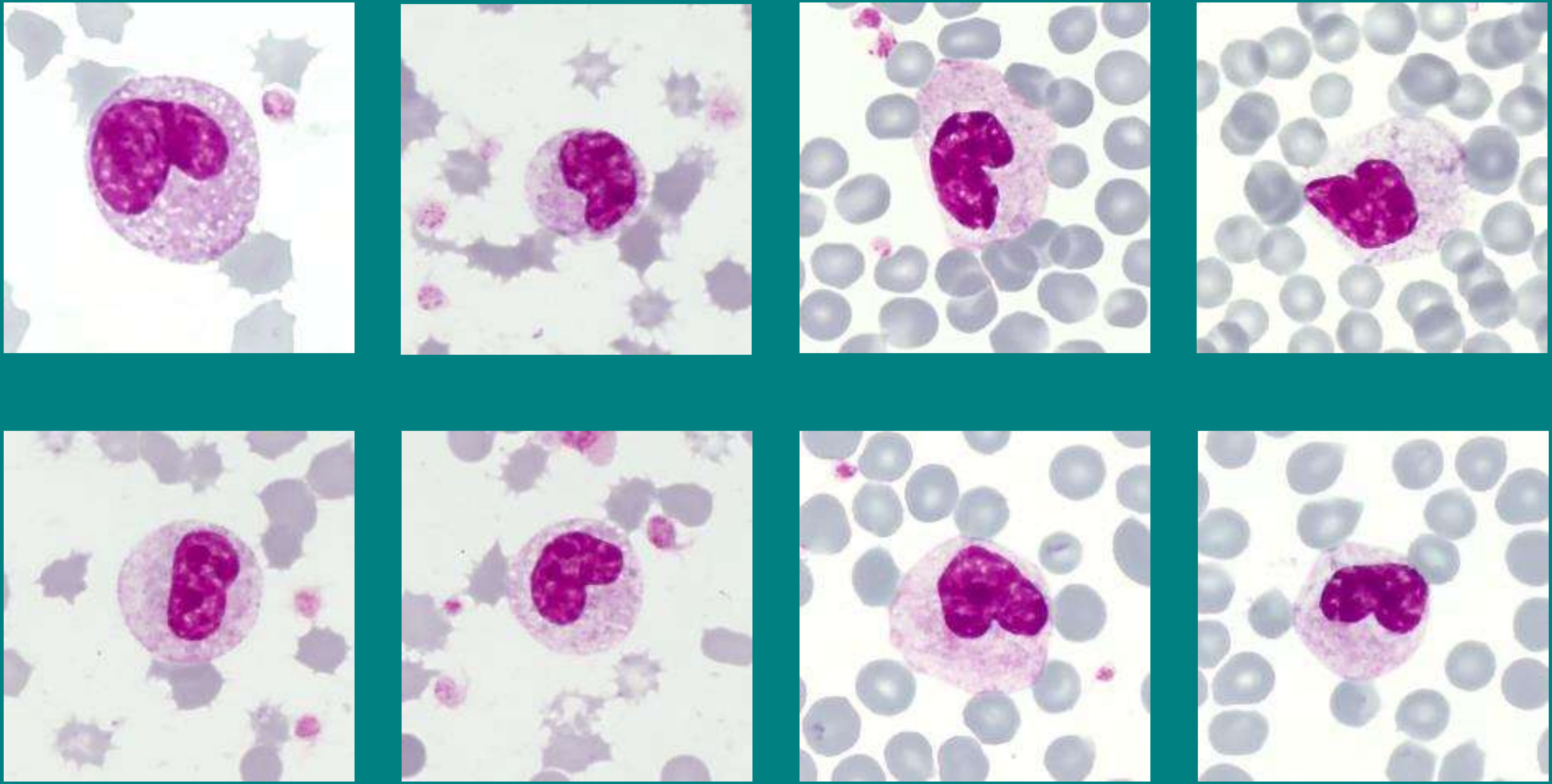

© M.G. Wagener, TiHo-Hannover, 2021

Myelocytes

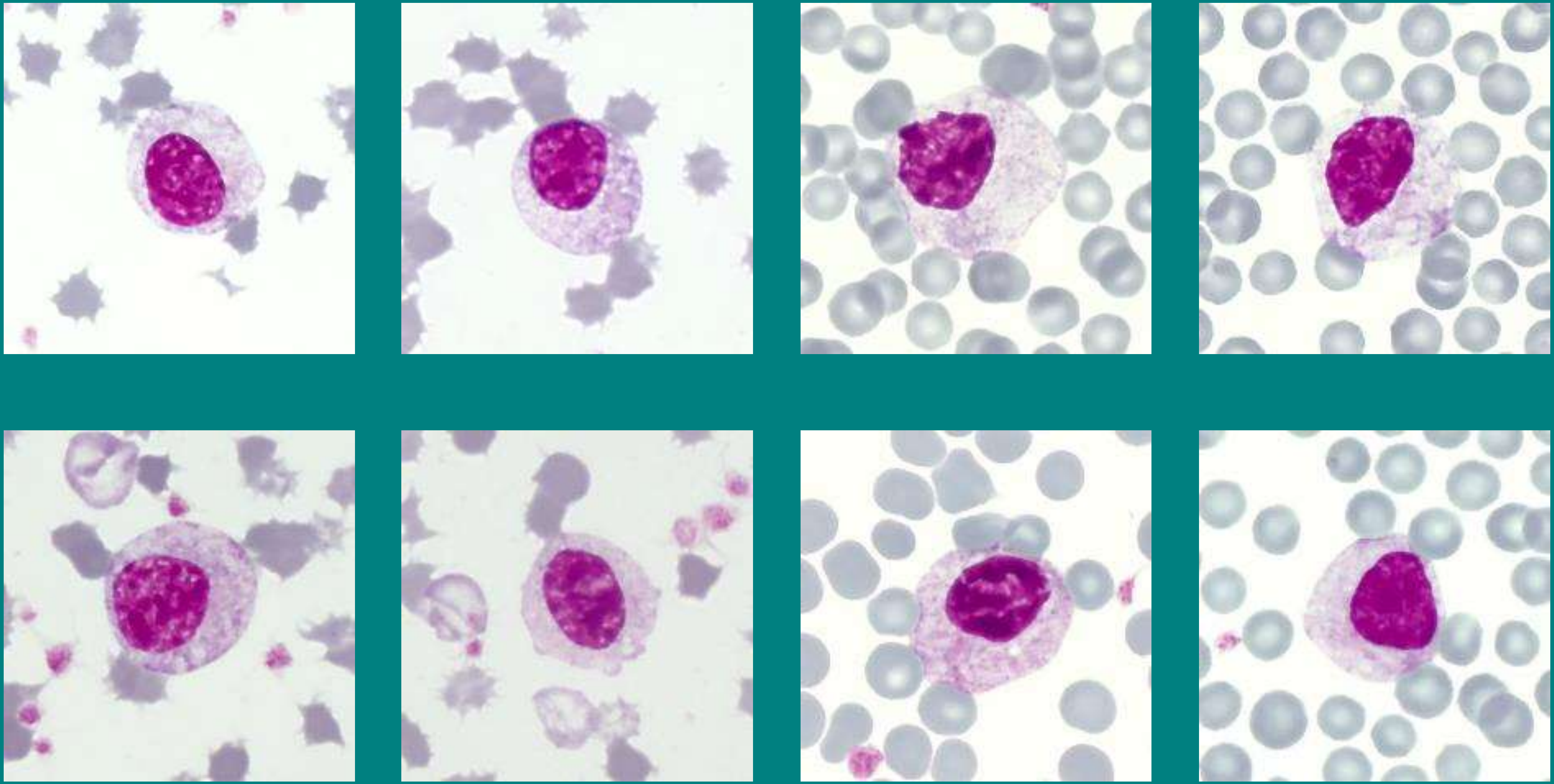

© M.G. Wagener, TiHo-Hannover, 2021
